# Epitranscriptomic mRNA modifications governing plant stress responses: underlying mechanism and potential application

**DOI:** 10.1111/pbi.13913

**Published:** 2022-09-09

**Authors:** Jianzhong Hu, Jing Cai, Tao Xu, Hunseung Kang

**Affiliations:** ^1^ Jiangsu Key Laboratory of Phylogenomics and Comparative Genomics, School of Life Sciences Jiangsu Normal University Xuzhou Jiangsu Province China; ^2^ Department of Applied Biology, College of Agriculture and Life Sciences Chonnam National University Gwangju Korea

**Keywords:** mRNA modification, m^6^A, stress response, epitranscriptomics, crop breeding

## Abstract

Plants inevitably encounter environmental adversities, including abiotic and biotic stresses, which significantly impede plant growth and reduce crop yield. Thus, fine‐tuning the fate and function of stress‐responsive RNAs is indispensable for plant survival under such adverse conditions. Recently, post‐transcriptional RNA modifications have been studied as a potent route to regulate plant gene expression under stress. Among over 160 mRNA modifications identified to date, N^6^‐methyladenosine (m^6^A) in mRNAs is notable because of its multifaceted roles in plant development and stress response. Recent transcriptome‐wide mapping has revealed the distribution and patterns of m^6^A in diverse stress‐responsive mRNAs in plants, building a foundation for elucidating the molecular link between m^6^A and stress response. Moreover, the identification and characterization of m^6^A writers, readers and erasers in *Arabidopsis* and other model crops have offered insights into the biological roles of m^6^A in plant abiotic stress responses. Here, we review the recent progress of research on mRNA modifications, particularly m^6^A, and their dynamics, distribution, regulation and biological functions in plant stress responses. Further, we posit potential strategies for breeding stress‐tolerant crops by engineering mRNA modifications and propose the future direction of research on RNA modifications to gain a much deeper understanding of plant stress biology.

## Graphical abstract

Post‐transcriptional RNA modifications, particularly N^6^‐methyladenosine (m^6^A), in mRNAs play a crucial role in plant stress responses, which could a potential strategy for breeding stress‐tolerant crops by engineering mRNA modifications.

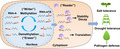

## Introduction

The global climate change has created conditions that are harmful environments to plants. Such environmental change can significantly impede plant growth and reduce crop yield worldwide. In particular, plants growing in altered environment often encounter abiotic stresses, such as drought, high salinity and extreme temperatures, as well as biotic stresses, such as insects, microorganisms and viruses. Being sessile, plants have evolved multitude of strategies to cope with the potentially destructive effects of these stressors and to survive in harmful environment. For instance, the expression of genes involved in stress responses is precisely reprogramed at the transcriptional and post‐transcriptional levels. An efficient pathway of post‐transcriptional gene regulation is the rapid modulation of the levels and status of existing RNAs in a stress‐dependent manner. Analogous to epigenetic regulation, which involves DNA methylation and histone modifications, a series of covalent RNA modifications has recently been elucidated as epitranscriptomic regulation; such regulation plays pivotal roles in shaping gene expression through the modulation of RNA metabolism at the post‐transcriptional level (Davalos *et al*., 2018). Recent bioinformatic and molecular evidence has illustrated the link between specific mRNA modifications and transcript abundances in plants under stress (Anderson *et al*., 2018; Cheng *et al*., 2021; He *et al*., 2021; Hou *et al*., 2022; Hu *et al*., 2021; Liu *et al*., 2020; Mao *et al*., 2021; Wang *et al*., 2022a; Yang *et al*., 2021; Zhang *et al*., 2021a, 2021b, 2021c). However, the precise mechanism underlying post‐transcriptional gene regulation via mRNA modification under stress awaits further investigation.

**Figure 1:**
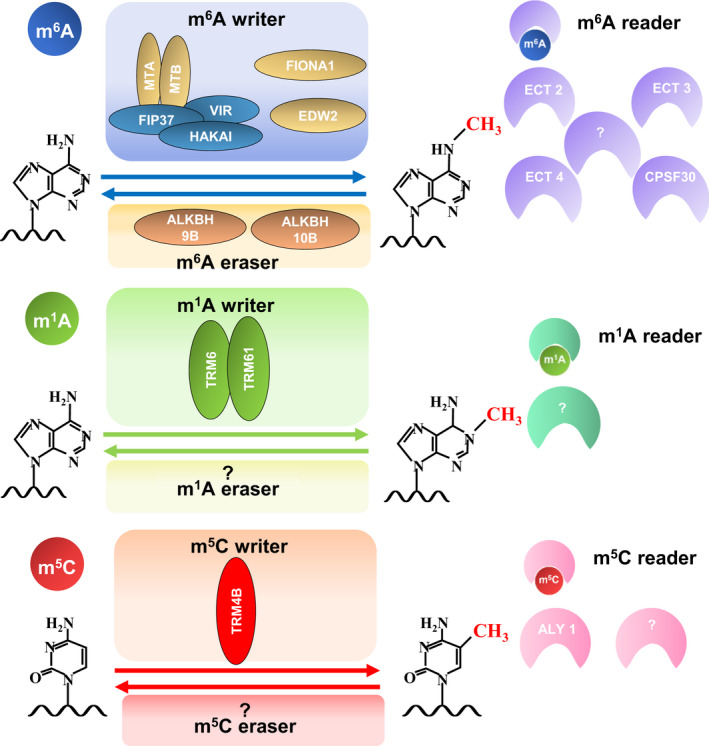
Cellular components involved in m^6^A, m^1^A and m^5^C modifications in plants. Characterized writers, erasers and readers that install, remove and interpret methylation marks, respectively, are shown. The MTA, MTB, FIP37, VIR and HAKAI m^6^A writers and the TRM6 and TRM61 m^1^A writers form complexes. Question marks (?) denote unidentified components.

Among over 160 diverse RNA modifications identified to date, N^6^‐methyladenosine (m^6^A), N^1^‐methyladenosine (m^1^A) and 5‐methylcytidine (m^5^C) are common and abundant internal modifications found in coding RNAs. Among these, m^6^A is the most prevalent and the best‐elucidated modification of eukaryotic mRNAs (Dominissini *et al*., 2012; Ke *et al*., 2015; Lence *et al*., 2016; Li *et al*., 2019; Linder *et al*., 2015; Luo *et al*., 2014; Meyer *et al*., 2012; Schwartz *et al*., 2013; Zhao *et al*., 2017). The cellular components involved in these modifications include methyltransferases (referred to as ‘writers’) and demethylases (referred to as ‘erasers’), which introduce and remove methylation marks, respectively, as well as RNA‐binding proteins (referred to as ‘readers’), which recognize and interpret the methylation marks (Davalos *et al*., 2018; Zaccara *et al*., 2019, Shi *et al*., 2019a; Figure 1). In particular, writer components responsible for m^6^A instalment on mRNA have been well characterized. MTA and MTB form heterodimers that are core elements displaying m^6^A methyltransferase activity, and three additional factors, including FIP37, VIR and HAKAI, form writer complexes that are either fully or partly required for m^6^A deposition (Růžička *et al*., 2017; Figure 1). Two additional m^6^A writers, FIONA1 and enhanced downy mildew2 (EDM2) that deposit m^6^A marks on a small group of mRNA, have recently been characterized (Figure 1; Ma *et al*., 2021; Wang *et al*., 2022b; Xu *et al*., 2022). Although a few m^6^A erasers and readers have been characterized so far (Figure 1), a recent genome‐wide analysis of m^6^A writers, erasers and readers in 22 plant species revealed that multiple family members of m^6^A writers, erasers and readers exist in plants and m^6^A machineries are highly conserved across plant species (Yue *et al*., 2019). However, the functions of these effectors are yet to be characterized. In both animals and plants, disruption of any of these cellular components results in abnormal development and altered stress responses (Jiang *et al*., 2021; Shao *et al*., 2021; Wilkinson *et al*., 2021). In animals, the roles of m^6^A, m^5^C and m^1^A in response to various stresses, including hypoxia, oxidative stress and UV radiation, have been demonstrated (Wilkinson *et al*., 2021). Moreover, transcriptome‐wide m^6^A and m^5^C profiling has been performed in diverse plant species under various abiotic and biotic stresses (Table 1). However, the functions and significance of these modifications in plant stress responses remain largely unexplored. Therefore, the key questions are why RNA modification patterns vary under specific stress conditions and how RNA modifications are associated with altered transcript and protein levels and cellular localization in response to stresses. The re‐localization of readers is associated with the stability and translation of transcripts under stress conditions. For instance, YTHDF2—a well‐characterized m^6^A reader in animals—is re‐localized from the cytoplasm to the nucleus under heat stress, which helps preserve m^6^A marks in the 5′‐untranslated region (UTR) by blocking the binding of the m^6^A eraser fat mass‐ and obesity‐association protein (FTO) and promoting the initiation of m^6^A‐mediated cap‐independent translation (Zhou *et al*., 2015). Furthermore, ECT2 (YTH9), a well‐characterized m^6^A reader in plants, is re‐localized from the cytoplasm to the stress granules (SGs) under heat shock (Scutenaire *et al*., 2018). Other interesting phenomena include the shift of m^6^A marks within the same transcript and the redistribution of m^6^A on new transcripts under stress. For instance, under salt stress, the abundance of global m^6^A modifications in the 5′‐ and 3′‐UTRs increases, and the introduction of m^6^A marks in the 5′‐UTR under salt stress is linked to the secondary structure and stability of mRNAs (Kramer *et al*., 2020). Therefore, the installation and interpretation of methylation marks on RNAs are crucial for stress adaptation in plants.

**Table 1:** List of m^6^A‐ and m^5^C‐seq in various plant species under different stresses

Modification	Stress type	Stress	Plant species	Plant tissues used for sequencing	References
m^6^A	Abiotic stress	Salt	Arabidopsis	Four‐week‐old rosette leaves	Anderson *et al*. (2018)
			Arabidopsis	Seven‐day‐old seedlings	Hu *et al.* (2021)
			Rice	Shoots, Roots	Wang *et al*. (2022a)
			Sweet sorghum	Roots	Zheng *et al*. (2021)
		Drought	Apple	One‐year‐old seedlings	Mao *et al*. (2021)
				Leaves	Hou *et al*. (2022)
			Sea buckthorn	Mix of Leaves, stems and roots	Zhang *et al*. (2021b)
		Heat	Pak‐choi	Five‐leaf‐stage seedlings	Liu *et al*. (2020)
		Low‐temperature	Tomato	Anthers	Yang *et al*. (2021)
		Cadmium	Rice	Roots from five‐day‐old seedlings	Cheng *et al*. (2021)
	Biotic stress	RSV, RBSDV	Rice	Seedlings	Zhang *et al*. (2021a)
		WYMV	Wheat	Reviving stage plants	Zhang *et al*. (2021b)
		CGMMV	Watermelon	Seedlings	He *et al*. (2021)
m^5^C	Abiotic stress	Heat	Rice	Three‐week‐old seedlings	Tang *et al*. (2020a, 2020b)

CGMMV, cucumber green mottle mosaic virus; RBSDV, rice black‐streaked dwarf virus; RSV, rice stripe virus; WYMV, wheat yellow mosaic virus.

In this review, we summarize recent progresses in our understanding of mRNA modifications, particularly m^6^A methylation, in plants under stress. Further, we discuss the stress‐induced dynamics, stress‐mediated distribution and roles of m^6^A modification in mRNA metabolism and the related mechanistic links with plant stress response. Finally, we suggest potential strategies for breeding stress‐tolerant crops by engineering mRNA modifications and propose future directions for research to gain a much deeper understanding of mRNA modifications in plant stress biology.

## Occurrence of mRNA modifications in plant stress response

Two mRNA modifications, namely m^6^A and m^5^C, have been mapped in plants under abiotic and biotic stresses (Table 1). In mammals, m^1^A, in addition to m^6^A and m^5^C, has been mapped and functionally characterized under stress (Alriquet *et al*., 2021). In *Arabidopsis*, m^5^C levels, as measured by liquid chromatography‐mass spectrometry decrease following exposure to drought or heat (Cui *et al*., 2017). Conversely, in rice, the global level of m^5^C, which regulates chloroplast function and reactive oxygen species (ROS) formation, increases under heat stress (Tang *et al*., 2020b). Bioinformatics analyses have revealed that several enzymes modifying tRNA bases, including m^5^C, m^1^A, m^7^G, Am and Cm, are potentially involved in stress response (Wang *et al*., 2017b). Further, Trm6/61 is responsible for the deposition of m^1^A on both tRNAs and mRNAs (Tang *et al*., 2020a; Yang *et al*., 2020). Similarly, m^7^G is installed on both tRNAs and mRNAs in plants (Chu *et al*., 2018). Notably, the levels of m^1^A and m^7^G changed differentially in *Arabidopsis* and rice under stress (Wang *et al*., 2017b). In the present review, we will focus on mRNA modifications associated with plant stress response through their effects on the base‐pairing capacity and stability of mRNAs. A previous study on mRNA modifications and turnover in *Arabidopsis* revealed that modifications were enriched in mRNAs responsive to various abiotic and biotic stresses (Vandivier *et al*., 2015). As such, these modifications were primarily enriched in uncapped and easily degrading mRNAs. The possible reasons for extensive deposition of modification marks on stress‐responsive mRNAs are as follows: (i) to maintain these stress‐responsive transcripts at a basal level during normal growth and development and (ii) to initiate alternative translation to rapidly respond to stresses. For instance, m^1^A is deposited around the start codon and affects Watson–Crick base pairing, which is positively correlated with alternative translation in response to stress in humans (Dominissini *et al*., 2016). These results imply a potential role of many other unidentified and uncharacterized mRNA modifications in plant stress response. However, mapping and characterization of these modifications are challenging due to their low abundance. Among the mRNA modifications listed above, m^6^A is the best‐elucidated one and the primary focus of the present review.

## Landscape and dynamics of m^6^A in plant response to diverse stresses

The mapping of mRNA modifications is a fundamental part of understanding how these modifications are dynamically regulated to modulate gene expression in stress response. At present, transcriptome‐wide mapping of mRNA modifications in plant stress response is at the initial stage, with m^6^A being the best‐characterized mRNA modification, followed by m^5^C. Recent advances in high‐throughput sequencing technologies, including methylated RNA immunoprecipitation sequencing (MeRIP‐seq), nanopore direct RNA sequencing, methylation individual‐nucleotide‐resolution cross‐linking and immunoprecipitation (miCLIP), and m^6^A‐selective chemical labelling (m^6^A‐SEAL), have improved the mapping of m^6^A landscape. Among these, MeRIP‐seq is the most widely used to map m^6^A in diverse plant species under various stresses (Table 1).

## m^6^A architecture in stress‐mediated methylomes in plants

Similar to the observations under normal growth and development in plants (Luo *et al*., 2014; Wan *et al*., 2015; Shen *et al*., 2016; Duan *et al*., 2017; Wang *et al*., 2017c; Miao *et al*., 2019; Parker *et al*., 2020; Hu *et al*., 2022) and other eukaryotes (Dominissini *et al*., 2012; Ke *et al*., 2015; Lence *et al*., 2016; Li *et al*., 2019; Linder *et al*., 2015; Meyer *et al*., 2012; Schwartz *et al*., 2013; Zhao *et al*., 2017), m^6^A is predominantly enriched at the 3′‐UTR and the near‐stop codon under stress (Anderson *et al*., 2018; Cheng *et al*., 2021; He *et al*., 2021; Hou *et al*., 2022; Hu *et al*., 2021; Liu *et al*., 2020; Mao *et al*., 2021; Wang *et al*., 2022a; Yang *et al*., 2021; Zhang *et al*., 2021a, 2021b, 2021c; Zheng *et al*., 2021). These findings indicate that m^6^A distribution on mRNAs is conserved across organisms under both normal and stress conditions. Analysis of the m^6^A sequence revealed an RR(m^6^A)CH (R = A/G; H = A/C/U) motif in all eukaryotes (Zhang *et al*., 2021b; Zheng *et al*., 2021) and a URU(m^6^A)Y (Y = C/U) motif unique to plants (Guo *et al*., 2021; Hu *et al*., 2021). Moreover, an AAACCV (V = U/A/G) motif in Pak‐choi (Liu *et al*., 2020) and a WKUAH (W = U/A; K = G/U) motif in rice (Ma *et al*., 2021) were detected under heat stress. The presence of multiple plant‐specific m^6^A motifs as well as the RR(m^6^A)CH motif conserved across all eukaryotes implies multifaceted functions of m^6^A modifications in plants. Notably, many stress‐responsive transcripts, including mRNAs involved in response to water stress, disease and oxidative stress, are enriched in m^6^A. The key question is the necessity and importance of m^6^A in plant stress response, which will be discussed subsequently.

## m^6^A dynamics during plant response to diverse stresses

Although the overall m^6^A patterns are conserved across plants, the global and individual m^6^A levels are dynamically regulated in response to diverse stresses. In fact, the global m^6^A levels increased under salt stress in *Arabidopsis* (Hu *et al*., 2021) and upon viral infection in rice (Zhang *et al*., 2021a) but decreased under drought stress in sea buckthorn (Zhang *et al*., 2021b), under moderate low‐temperature stress in tomato (Yang *et al*., 2021) and upon cucumber green mottle mosaic virus (CGMMV) infection in watermelon (He *et al*., 2021). In contrast, global m^6^A levels remain unchanged in apples exposed to drought stress (Hou *et al*., 2022; Mao *et al*., 2021), Pak‐choi exposed to heat stress (Liu *et al*., 2020), rice exposed to heavy metal (cadmium) stress (Cheng *et al*., 2021), and wheat infected by yellow mosaic virus (Zhang *et al*., 2021c). These findings indicate dynamic m^6^A modifications in a stress‐ or species‐specific manner. In this context, a fundamental question is how various stresses differentially regulate m^6^A levels in plants, and the most plausible answer to this is the altered expression of m^6^A writers or erasers under stress. Salt stress induced the expression of several m^6^A writers (e.g. *MTA*, *MTB* and *VIR*), subsequently augmenting m^6^A levels in *Arabidopsis* (Hu *et al*., 2021). Moreover, drought stress induced the expression of the m^6^A eraser *ALKBH10B* homologues, thereby suppressing m^6^A levels in sea buckthorn (Zhang *et al*., 2021b). In addition, cucumber green mottle mosaic virus infection increased the expression of *ClALKBH4B*, presumably reducing the global m^6^A levels in watermelon (He *et al*., 2021). These findings indicate the differential regulation of m^6^A effectors under various stresses.

Because the global m^6^A levels are determined by the expression level of m^6^A writers or erasers, it would be worth exploring whether specific stress regulates the expression of either writers or erasers or regulates the expression of both writers and erasers in a plant.

Although the global abundance of m^6^A remains unchanged in plants under the diverse stresses described above, transcriptome‐wide analysis of stress‐mediated m^6^A methylomes has revealed a dynamic feature of stress‐induced redistribution of m^6^A on mRNAs. Salt stress increases m^6^A deposition in the 5′‐ and 3′‐UTRs but not in the coding regions in *Arabidopsis* (Hu *et al*., 2021). Moreover, many transcripts with salt‐specific m^6^A marks are linked to salt or osmotic stress (Anderson *et al*., 2018; Hu *et al*., 2021). The next key question is the biological significance of these salt stress‐specific m^6^A marks in stress response. A plausible hint can be obtained from recently reported m^6^A methylomes in salt‐tolerant and salt‐sensitive genotypes of sweet sorghum. As such, the salt‐sensitive genotype presented considerable m^6^A redistribution in response to salt stress, while the salt‐tolerant genotype exhibited little changes in m^6^A patterns (Zheng *et al*., 2021). Similarly, m^6^A patterns in the shoots and roots of a salt‐tolerant rice cultivar remain unaltered under salt stress (Wang *et al*., 2022a). These findings suggest that m^6^A redistribution is an adaptive response of salt‐sensitive plants to saline environment. Importantly, the comparison of m^6^A marks between salt‐sensitive and salt‐tolerant genotypes revealed m^6^A deposition in the former under salt stress, but not under normal conditions, was similar to that in the latter in the presence or absence of salt stress (Zheng *et al*., 2021). These observations suggest that the salt‐induced m^6^A modifications in salt‐sensitive genotype are resistant marks found in the salt‐tolerant genotype during long‐term salt acclimation, implying a functionally crucial role of these stress‐induced m^6^A marks in salt stress response. Thus, the regulation of m^6^A dynamics appears to be an evolved regulatory strategy of plant adaptation to stress. This leads us to contemplate another interesting point of whether and how m^6^A modifications can serve as prime marks for the activation of stress‐responsive genes, similar to the function of DNA methylation and histone modifications under stress. Additional comparative analyses of m^6^A patterns between stress‐sensitive and stress‐tolerant genotypes, particularly in crops, would be valuable to further understand the nature and roles of m^6^A dynamics in stress adaptation.

## Position‐ and transcript‐specific m^6^A dynamics in response to stress

An important question is whether the m^6^A marks are installed in a position‐ and transcript‐specific manner. Although every transcript contains many potential RRACH motifs throughout the sequence, transcriptome‐wide m^6^A profiling has revealed that only one or two of these potential motifs are methylated in a vast majority of transcripts in both plants and other eukaryotes. As described above, various stresses can alter the m^6^A dynamics and distribution within the same or new transcripts, indicating that specific RRACH motifs are selected for m^6^A modification in a position‐ and transcript‐specific manner depending on environmental cues and developmental stages. An intriguing question is what drives the selection of RRACH sites for m^6^A methylation under specific stress conditions. Several studies in mammals have implicated histone modifications, transcription factors, specific RNA‐binding proteins and RNA polymerase II in selective m^6^A modification in a transcript (Zaccara *et al*., 2019). For instance, the histone modification H3K36me3 interacts with METTL14 to anchor the writer complex to a particular region of nascent RNA for m^6^A methylation (Huang *et al*., 2019), and the transcription factors, SMAD2/3, recruit the METTL3/14‐WTAP complex to the promoter region for transcript methylation in response to transforming growth factor‐beta signal (Bertero *et al*., 2018). Further, the RNA‐binding motif protein 15 (RBM15) and RBM15B interact with the m^6^A methylation complex and guide the writer complex to a specific mRNA for m^6^A methylation (Patil *et al*., 2016), and RNA polymerase II interacts with METTL3 to mediate m^6^A modification in newly transcribed RNAs (Slobodin *et al*., 2017). H3K36me2, rather than H3K36me3, is crucial for m^6^A deposition in plants (Shim *et al*., 2020) and that MTA (plant orthologue of METTL3) likely interacts with RNA polymerase II in *Arabidopsis* (Bhat *et al*., 2020). Although there is no evidence supporting the existence of similar mechanisms in plant response to stresses, histone modifications and transcription factors most likely regulate stress response through crosstalk with the m^6^A modification pathway. In addition to guiding the m^6^A writers to a specific site within the transcript, the possible involvement of m^6^A erasers cannot be overruled. As m^6^A methylation is processed co‐transcriptionally, certain transcripts may be methylated at multiple sites in their nascent RNAs and some of these methylation marks may be selectively removed by m^6^A erasers depending on the developmental stage or stress conditions. Discovery of these mechanisms will help ascertain the significance of stress‐induced m^6^A dynamics and the sequence context‐specificity of m^6^A modifications in stress responses.

## Molecular roles of m^6^A in mRNA metabolism in plant stress response

Numerous studies have demonstrated the vital roles of m^6^A modifications in mRNA metabolism, including mRNA stability (Anderson *et al*., 2018; Duan *et al*., 2017; Kramer *et al*., 2020; Wei *et al*., 2018), intron splicing (Ma *et al*., 2021), translation (Guo *et al*., 2021; Huang *et al*., 2021; Luo *et al*., 2020; Luo *et al*., 2021; Shao *et al*., 2021; Zhou *et al*., 2021), alternative polyadenylation (APA; Hou *et al*., 2021; Hu *et al*., 2021; Song *et al*., 2021), secondary structure formation (Kramer *et al*., 2020) and transcriptome integrity (Pontier *et al*., 2019). Typically, regulation of RNA metabolism of an individual transcript is associated with the location of m^6^A marks. For instance, m^6^A located in the 3′‐UTR and near the stop codon is primarily involved in modulating transcript stability (Hou *et al*., 2021; Luo *et al*., 2020; Zhou *et al*., 2019, 2021) and transcriptome integrity (Pontier *et al*., 2019). m^6^A present in the 5′‐UTR is involved in the regulation of translation (Hou *et al*., 2022), and m^6^A in the coding region affects the stability and splicing of mRNA (Ma *et al*., 2021; Zhou *et al*., 2021).

**Figure 2:**
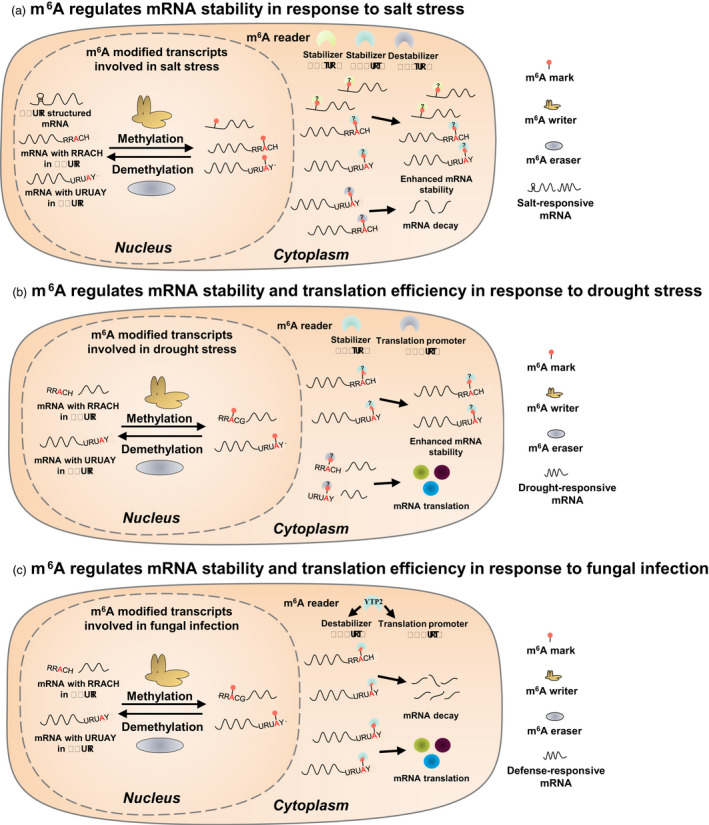
Regulatory roles of m^6^A in mRNA metabolism in plant stress responses, as determined by the analysis of writer, eraser or reader mutants. (a) Under salt stress, m^6^A in the 3′‐UTR can either increase or decrease the stability of salt‐responsive mRNAs and m^6^A in the 5′‐UTR can affect RNA secondary structure, thereby positively regulating the stability of salt‐responsive mRNAs. The m^6^A readers contributing to RNA stability must be verified. (b) Under drought stress, m^6^A in the 3′‐UTR can affect the stability of drought‐responsive mRNAs and m^6^A in 5′UTR can promote the translation efficiency of drought‐responsive mRNAs. The m^6^A readers contributing to RNA stability must be verified. (c) In fungal infection, the m^6^A reader binds the m^6^A mark in the 3′‐UTR to promote the degradation or translation of defence‐related mRNAs.

The location‐specific m^6^A function on mRNA metabolism is associated with the binding of specific m^6^A readers. For instance, m^6^A located in the 3′‐UTR can be recognized by a reader protein (e.g. YTHDF2) that recruits deadenylase complex (CCR4–NOT) to destabilize mRNA (Du *et al*., 2016), while m^6^A located in the 5′‐UTR can be recognized by a reader protein (e.g. eIF3) to facilitate translation (see below). Although our understanding of the roles of m^6^A in mRNA metabolism under stress is severely limited, its involvement in the regulation of mRNA stability and translation is relatively well‐elucidated thus far (Figure 2) and is discussed in depth below.

### m^6^A regulates mRNA stability in plant response to abiotic and biotic stresses

Integrated application of m^6^A‐seq and RNA‐seq is a reliable approach to explore the association of m^6^A levels with mRNA abundance in plants exposed to various stresses. In *Arabidopsis*, the deposition of m^6^A marks in salt‐responsive genes under salt stress is associated with transcript stabilization, due presumably to the reduced endonucleolytic cleavage around the m^6^A site (Anderson *et al*., 2018). Moreover, salt‐specific m^6^A deposition reduced mRNA secondary structures under salt stress, ultimately increasing mRNA stability and protein levels (Kramer *et al*., 2020). The m^6^A writer *vir* mutant with reduced m^6^A levels exhibited a lower abundance of mRNA under salt stress, suggesting that m^6^A marks protect transcripts from degradation (Hu *et al*., 2021). However, the abundance of other m^6^A‐modified transcripts increased with decreased m^6^A levels in the *vir* mutant, suggesting a negative correlation between m^6^A and transcript stability under salt stress (Hu *et al*., 2021). Notably, the instability of m^6^A‐modified transcripts is associated with APA (Hu *et al*., 2021). Contrary to either increased or decreased levels of mRNAs with reduced m^6^A marks under salt stress, the levels of m^6^A‐modified transcripts were biasedly increased in both *vir* and *fip37* mutants during development (Hu *et al*., 2021; Shen *et al*., 2016). These findings suggest that the m^6^A‐mediated control of mRNA stability is more complex during stress adaptation than during development. As m^6^A readers can modulate the degradation and stability of m^6^A‐modified mRNAs, stress‐specific m^6^A readers must be discovered and characterized to further elucidate the multifaceted roles of m^6^A marks in regulating mRNA stability under diverse stress conditions.

The correlation between m^6^A modification and transcript levels in crop species under stress has been recently explored. Interestingly, salt‐induced m^6^A modification was associated with increased transcript levels in salt‐sensitive but not salt‐tolerant genotypes of sweet sorghum (Zheng *et al*., 2021), whereas m^6^A modification was not associated with increased transcript levels in the shoots and roots of a salt‐tolerant rice genotype exposed to salt stress (Wang *et al*., 2022a). Furthermore, a positive correlation between m^6^A methylation and gene expression was observed in sea buckthorn under drought stress (Zhang *et al*., 2021b). In apple, the stability and abundance of transcripts encoding drought‐, lignin biosynthesis‐ and H_2_O_2_ pathway‐related genes increased under drought stress (Hou *et al*., 2022). Conversely, in tomato anther, low‐temperature‐induced m^6^A modification around the stop codon or in the 3′‐UTR was negatively correlated with transcript abundance (Yang *et al*., 2021). Meanwhile, no clear correlation between m^6^A modification and mRNA abundance was noted in Pak‐choi seedlings exposed to heat stress (Liu *et al*., 2020) and in rice subjected to heavy metal (cadmium) stress (Cheng *et al*., 2021). Notably, different effects of m^6^A modification on gene expression levels were observed in crops upon viral infection. For instance, a negative correlation between m^6^A methylation and transcript abundance was observed in watermelon infected with cucumber green mottle mosaic virus (He *et al*., 2021), but either positive or negative correlation between m^6^A methylation and gene abundance was noted in rice infected with rice stripe virus or rice black‐streaked dwarf virus (RBSDV) depending on the location of m^6^A in the transcript (Zhang *et al*., 2021a). These observations suggest that the effects of m^6^A on transcript abundance in crops cannot be generalized and can vary in a stress‐, species‐ or sequence‐dependent manner. Notably, however, these studies were based on m^6^A methylomes obtained from mock‐ or stress‐treated wild‐type plants. Considering that transcript stability can be affected by other factors, such as RNA‐binding proteins, microRNAs and transcription factors, the correlation between m^6^A and transcript abundance must be analysed in the mutants of m^6^A writers, erasers and readers to unveil the precise roles of m^6^A in the regulation of mRNA stability in plant response to abiotic and biotic stresses.

### m^6^A potentially regulates mRNA translation in plant response to stresses

Although the effects of m^6^A on translational control under stress remain largely unexplored, some plausible evidence allows us to speculate its potential role. For instance, in *Arabidopsis*, ECT2—a cytoplasm‐localized m^6^A reader—is re‐localized to SGs under heat stress (Scutenaire *et al*., 2018). As SGs are assembled under stress conditions and function to block translation by storing the mRNA‐ribosome complexes, SG‐localized ECTs may serve distinct functions in the regulation of translation under stress. ECT2 and ECT4, but not ECT3, are re‐localized to P‐bodies under osmotic stress (Arribas‐Hernández *et al*., 2018). P‐body is a membraneless biomolecular condensate involved in RNA degradation. Increasing evidence in human studies indicates that YTHDF2 binds to the transcripts re‐localized from translating pools to P‐bodies (Lee *et al*., 2020), implying that re‐localization of m^6^A readers from the cytosol to P‐bodies might be associated with osmotic stress response. Of note, under heat stress, YTHDF2—a well‐characterized m^6^A reader in humans—shows re‐localization from the cytosol to the nucleus, during which the m^6^A mark in 5′‐UTR is preserved through blocking the binding of the m^6^A eraser FTO, leading to the initiation of m^6^A‐mediated cap‐independent translation (Zhou *et al*., 2015). This distinct localization of YTH domain proteins in plants and humans indicates their functional diversity in different organisms under stress. Further, m^6^A modification in the 5′‐UTR improves the translation efficiency of drought‐related transcripts in apple, suggesting a positive role of m^6^A in the regulation of translation under drought stress (Hou *et al*., 2022). However, the mechanisms underlying m^6^A‐mediated translational control under stress remain elusive. Several lines of evidence obtained from studies in humans indicate the pivotal functions of m^6^A readers. For instance, m^6^A methylation in the 5′‐UTR serves as a selective mark for the binding of m^6^A readers, such as human YTHDF1, together with the eukaryotic translation initiation factor 3 (eIF3), to initiate cap‐independent translation under stress (Wang *et al*., 2015). In addition, the m^6^A writer METTL3 facilitates cap‐dependent translation through the recruitment of eIF3 to the translation initiation complex, with m^6^A marks serving as ribosome engagement sites, eventually promoting cap‐independent translation (Wang *et al*., 2015). Furthermore, OsMTA2—a rice orthologue of human METTL3—interacts with eIF3 to modulate growth and pollen development in rice by affecting the global translation status (Huang *et al*., 2021). These reports suggest that m^6^A methylation plays a crucial role in translational control in plants and animals under both normal and stress conditions. Nonetheless, further in‐depth mechanistic studies are warranted to identify the specific m^6^A readers or writers involved in translational control under different stress conditions.

## Biological roles of m^6^A in plant stress response

**Figure 3:**
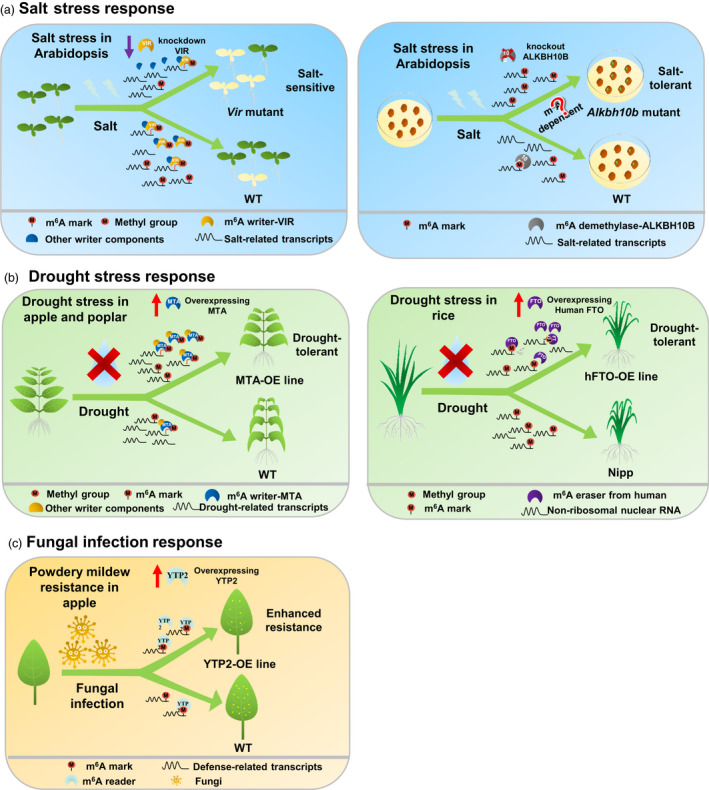
Biological functions of m^6^A writers, erasers and readers in plant stress response. (a) In *Arabidopsis*, the m^6^A writer VIR and the m^6^A eraser ALKBH10B regulate seedling growth and seed germination under salt stress. (b) In apple, poplar and rice, the m^6^A writer MTA and the human eraser FTO regulate drought tolerance. (c) In apple, the m^6^A reader YTP2 regulates powdery mildew resistance.

Following the identification and mapping of m^6^A methylomes in diverse plant species subjected to stress treatments, studies exploring the biological significance of m^6^A modifications in plant responses to abiotic and biotic stresses through the identification and characterization of m^6^A writers, readers and erasers in *Arabidopsis* and model crops are emerging (Figure 3), as discussed in depth below.

### Significance of m^6^A in plant response to abiotic stresses

To date, the significance of m^6^A in abiotic stress response has primarily been evaluated in *Arabidopsis*. Several recent studies investigating m^6^A writer and eraser mutants have shown that m^6^A is positively correlated with salt tolerance. For instance, VIR‐mediated m^6^A methylation regulates salt stress response by negatively modulating the abundance of transcripts encoding the negative effectors of salt stress (Hu *et al*., 2021), and knockout of ALKBH10B—an m^6^A eraser in *Arabidopsis*—promotes seed germination and seedling growth under salt or osmotic stress by down‐regulating m^6^A‐modified transcripts encoding the negative effectors of salt stress (Shoaib *et al*., 2021; Tang *et al*., 2021). Recently, ALKBH6 and ALKBH8B—the putative RNA demethylases in *Arabidopsis*—were shown to contribute to salt, drought or heat tolerance (Huong *et al*., 2020, 2022). Collectively, these results indicate a positive role of m^6^A modifications in salt tolerance. Notably, bioinformatic analysis of m^6^A writer, eraser and reader expression levels revealed that m^6^A writer or eraser expression levels were altered only marginally following stress treatment, although m^6^A reader expression levels were altered markedly under different stresses (Hu *et al*., 2019), suggesting their important roles in stress response. Before the discovery of ECT family proteins as m^6^A readers, ECT1 and ECT2 were implicated in calcium signalling in response to various external stimuli through their interaction with the stress response protein Calcineurin B‐Like‐Interacting Protein Kinase1 (Ok *et al*., 2005). Re‐localization of ECT2 to SGs under heat stress (Scutenaire *et al*., 2018) as well as of ECT2 and ECT4 to P‐bodies under osmotic stress (Arribas‐Hernández *et al*., 2018) strongly supported the notion that m^6^A is closely linked to abiotic stress response. Interestingly, ectopic expression of apple MhYTP2 in *Arabidopsis* enhanced tolerance to multiple abiotic stresses (Wang *et al*., 2017a), and MhYTP2 was recently identified as an m^6^A reader in apple (Guo *et al*., 2021 **).**

Furthermore, biological roles of m^6^A modifications in abiotic stress response have been explored in several crop species. For instance, PtrMTA overexpression enhanced the drought tolerance of *Populus* by altering trichome and root development (Lu *et al*., 2020). In apple, MdMTA played a positive role in drought tolerance by improving lignin deposition and ROS scavenging (Hou *et al*., 2022). In rice, the cadmium‐induced abnormal root development was associated with differential m^6^A modifications in roots (Cheng *et al*., 2021). Comprehensive expression analysis of genes encoding YTH domain proteins in rice and wheat revealed that *OsYTHs* and *TaYTHs* were up‐regulated under various abiotic stresses (Hu *et al*., 2019; Sun *et al*., 2020). Recent genome‐wide sequence and expression analyses of ALKBH family proteins in sugar beet revealed that BvALKBH10B—an orthologue of *Arabidopsis* AtALKBH10B—was significantly induced by salt stress, suggesting its involvement in salt stress response (Cui *et al*., 2022). Although accumulating evidence clearly indicates the cruciality of m^6^A modifications in crop responses to abiotic stresses, the cellular and mechanistic roles of m^6^A writers, erasers and readers await further characterization.

### Significance of m^6^A in plant response to biotic stresses

Unfortunately, only a few studies have demonstrated the involvement of m^6^A in biotic stress response in *Arabidopsis*. For instance, ALKBH9B affects the accumulation and systemic invasion of alfalfa mosaic virus by interacting with its coat protein, thereby influencing the viral infection cycle (Martínez‐Pérez *et al*., 2017, 2021). In *Arabidopsis*, the ENHANCED DOWNY MILDEW 2 (EDM2) has been characterized to be a specific gene conferring resistance against *Hyaloperonospora parasitica* by regulating the expression of RECOGNITION OF PERONOSPORA PARASITICA 7 (Eulgem *et al*., 2007). Recently, EDM2 was identified as a novel mRNA m^6^A writer in rice (Ma *et al*., 2021). However, given the lack of a typical methyltransferase domain in its structure, whether *Arabidopsis* EDM2 regulates m^6^A methylation remains unclear. Cleavage and polyadenylation specificity factor 30 (CPSF30)—a newly confirmed YTH domain‐containing m^6^A reader (Hou *et al*., 2021; Song *et al*., 2021)—confers resistance against *Pseudomonas syringae* through the basal and *R*‐gene‐mediated disease resistance pathways (Bruggeman *et al*., 2014); however, whether this defence response is m^6^A‐dependent remains unknown.

Several lines of evidence link m^6^A abundance with biotic stress responses of crops. For instance, in *Nicotiana tabacum*, tobacco mosaic virus (TMV) infection suppresses the expression of m^6^A methylase and demethylase orthologous genes, thereby decreasing m^6^A levels (Li *et al*., 2018b). However, the precise roles of m^6^A modifications in TMV resistance and host–virus interactions warrant further verification. Moreover, rice stripe virus and RBSDV infection significantly increased the global levels of m^6^A and altered the transcription of genes encoding m^6^A machinery, suggesting the involvement of m^6^A modification in defence response (Zhang *et al*., 2021a). Moreover, decreasing m^6^A levels in small brown planthopper (SBPH)—a vector of RBSDV—through the interference of two m^6^A writers, namely LsMETTL3 and LsMETTL14, significantly increases the RBSDV titres in the midgut cells of SBPH, suggesting a negative correlation between global m^6^A levels and virus replication (Tian *et al*., 2021). In addition, silencing of an m^6^A writer (PoIME1), eraser (PoALBK1) or reader (PoYTH1 and PoYTH2) in *Pyricularia oryzae* revealed the potential correlation between m^6^A level and virulence in rice (Shi *et al*., 2019b). These findings indicate that the regulation of m^6^A dynamics may be a potential strategy to control virus replication and infection in the host. As such, MhYTP2 overexpression enhanced the resistance of apple to powdery mildew by decreasing the stability of Mildew Locus O 19 mRNA and promoting the translation efficiency of antioxidant genes (Guo *et al*., 2021). Comparative analysis of m^6^A profiles in wheat without or with wheat yellow mosaic virus infection revealed many transcripts involved in defence response and plant‐pathogen interactions, suggesting the potential roles of m^6^A in pathogen resistance (Zhang *et al*., 2021c). Furthermore, CGMMV infection in watermelon altered the levels of m^6^A and transcripts involved in plant immunity, suggesting a possible regulatory role of m^6^A against this virus (He *et al*., 2021). Although these findings highlight the involvement of m^6^A in plant responses to biotic stress, the viral infection stage at which m^6^A functions and the molecular mechanisms underlying m^6^A‐mediated viral defence remain unexplored.

## Potential strategies for engineering mRNA modification to develop stress‐tolerant crops

**Figure 4:**
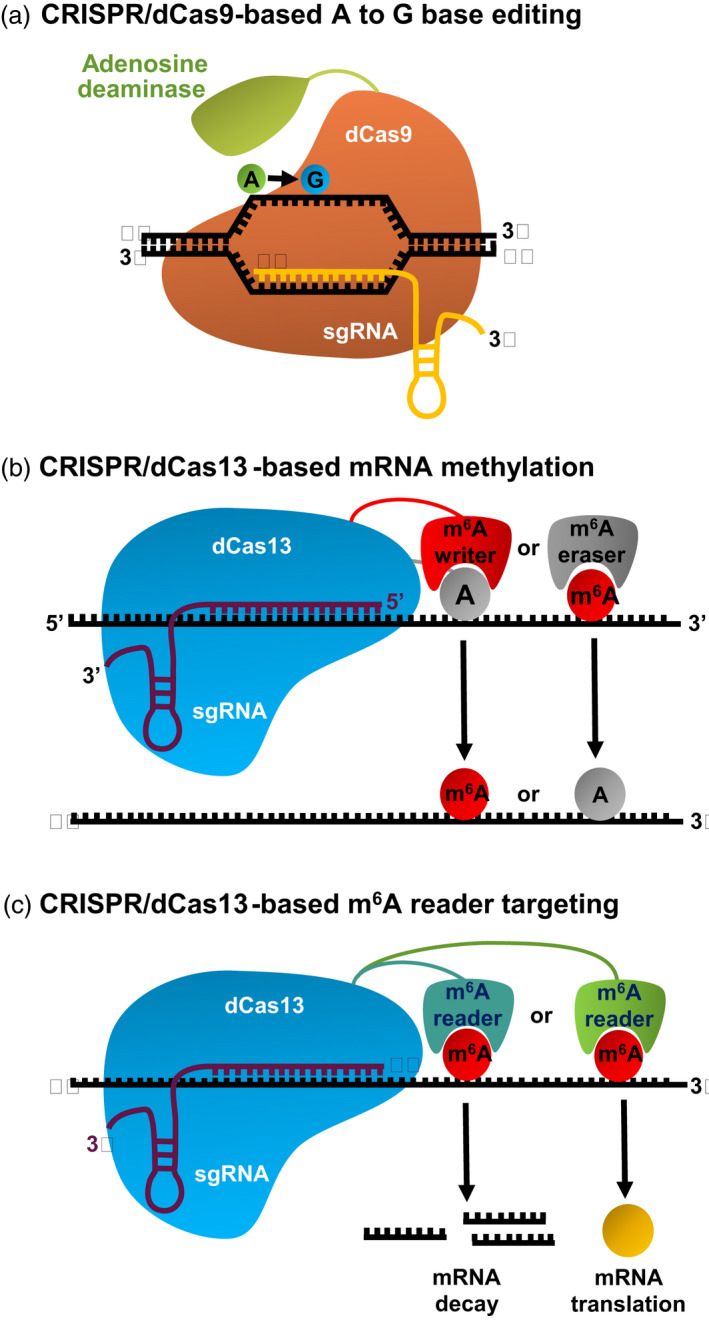
Potential strategies for engineering mRNA modifications. (a) CRISPR‐dCas9‐adenosine deaminase fusion can be used to edit A to G in genomic DNA, resulting in the removal of a potential m^6^A site in mRNA. (b) CRIPSR‐dCas13‐m^6^A writer or –m^6^A reader fusion can directly add or remove the target m^6^A mark in a specific mRNA. (c) CRISPR‐dCas13‐m^6^A reader fusion can modulate the interpretation of m^6^A marks, thereby altering mRNA degradation or translation.

Engineering mRNA modifications holds a great potential to improve plant tolerance of different environmental stresses due to their insignificant effects on plant genome integrity but high efficiency in the regulation of mRNA abundance. A recent study demonstrated an exciting and successful application of m^6^A modification to improve crop productivity and stress tolerance (Yu *et al*., 2021). Briefly, ectopic expression of FTO, an m^6^A demethylase in humans, in rice and potato not only considerably increased the biomass and yield by approximately 50% in the field trial but also enhanced plant tolerance of drought stress (Figure 3). Of note, however, although that study underscored the great potential of engineering m^6^A erasers for crop improvement, modification of overall m^6^A by engineering m^6^A writers or erasers may induce undesirable side effects, which should be closely monitored. Therefore, to overcome those seemingly undesirable side effects, more precise manipulation of m^6^A within a specific transcript is warranted. Here, we propose three potential approaches to engineer mRNA methylation for crop improvement (Figure 4). First, a specific adenosine (A) in the genomic DNA can be modified to guanosine (G) using CRISPR‐Cas‐derived base editors, such as the adenine base editor, generated by fusing the catalytically impaired Cas9 protein with signal‐stranded DNA‐specific adenosine deaminase (Gao, 2021). This A to G base editing system mimics the demethylase function to disrupt modification at a specific m^6^A site, eventually modulating the target mRNA abundance. This approach is particularly useful when the gene of interest is essential for plant development and its knockout can be lethal. Second, specific mRNA at a particular site can be modified using either a CRISPR‐Cas9‐ or CRISPR‐Cas13‐based system targeting RNA methylation. This system is generated by fusing dCas9 or dCas13 with either methyltransferase or demethylase to directly add or remove modification marks in the target endogenous RNA molecules without altering the nucleotide sequence of genomic DNA (Li *et al*., 2020; Liu *et al*., 2019; Wilson *et al*., 2020). Third, the reader‐mediated interpretation of modified mRNA can be regulated using the CRISPR‐Cas13b‐fused reader approach. For instance, the programmable dPspCas13b‐m^6^A reader protein system, in which the human m^6^A reader proteins YTHDF1 and YTHDF2 are fused to a catalytically inactive PspCas13b protein, can target the reader to a specific m^6^A site within a specific mRNA using guide RNA complementarity (Rauch *et al*., 2018). To date, the first strategy has been successfully utilized in plants (Li *et al*., 2018a; Zong *et al*., 2017), while the last two techniques are yet to be optimized in plants. Further advances in these techniques for plants are expected to substantially boost the application of epitranscriptomics for crop improvement.

## Concluding remarks and future perspectives

At present, the elucidation of mRNA modifications in plant responses to stress is only at the nascent stage. Except those of m^6^A, the biological functions of many mRNA modifications, such as m^5^C and m^1^A, await further exploration. Although accumulating evidence has highlighted the involvement of m^6^A modifications in stress response, many questions, such that how m^6^A is added to or removed from mRNA transcripts in a stress‐dependent manner and how m^6^A differentially regulates the abundance of stress‐responsive transcripts under different stress conditions, remain unresolved. In addition, majority of the m^6^A maps reported thus far were created using antibody‐based MeRIP‐seq data, which do not specify the precise location of m^6^A site in a transcript. Therefore, cutting‐edge sequencing modalities, such as nanopore direct RNA‐seq (Parker *et al*., 2020; Pratanwanich *et al*., 2021), MAZTER‐seq (Garcia‐Campos *et al*., 2019), m^6^A‐REF‐seq (Zhang *et al*., 2019) and miCLIP‐seq (Linder *et al*., 2015), should be applied to map m^6^A modifications at a single‐base resolution, which would enable the determination of the precise stoichiometry and dynamics of specific m^6^A modifications under different stress conditions. Furthermore, analysis of stress‐induced m^6^A methylomes is crucial for distinguishing the ‘constitutive’ and ‘dynamic’ modulations associated with stress, which will provide valuable information to precisely engineer RNA modifications for crop improvement. With recent breakthroughs in CRISPR genome editing techniques in animals and plants, the application of base editing systems that can modify single bases and the CRISPR‐Cas13‐based targeting RNA methylation system is anticipated greatly accelerate epitranscriptomic studies aimed at improving crop yield and stress tolerance. Moreover, considering that writers, erasers and readers responsible for the installation, removal and interpretation of different RNA modifications in crops remain largely elusive, these components must be identified and characterized to aid the engineering of specific RNA modifications for improving agriculturally important traits of crops.

In addition to elucidating the roles of m^6^A in the regulation of mRNA metabolism, whether and how m^6^A modifications regulate the fate of non‐coding RNAs (ncRNAs), including microRNAs (miRNAs) and long non‐coding RNA (lncRNAs), in plants under stress is of great interest. miRNAs are important players in plant stress response (Shriram *et al*., 2016). Likewise, lncRNAs are expressed in a stress‐specific manner and involved in stress response (Quinn and Chang, 2016; Jia *et al*., 2020). In mammals and plants, m^6^A marks deposited on miRNAs and lncRNAs affect miRNA biogenesis (Alarcón *et al*., 2015; Bhat *et al*., 2020; Wu *et al*., 2018) and lncRNA function (Liu *et al*., 2021; Xu *et al*., 2021; Yang *et al*., 2018). Therefore, how m^6^A regulates the biogenesis and functions of miRNAs and lncRNAs in plant response to stresses is worth exploring. Exploiting the m^6^A‐mediated regulation of these ncRNAs will expand our repertoire of epitranscriptomic modulations related to plant stress tolerance.

In summary, a rapid progress in transcriptome‐wide mapping has enabled the unveiling of the regulatory roles of m^6^A modifications in plant responses to diverse abiotic and biotic stresses. Notwithstanding, many challenges remain in identifying and characterizing the cellular components of writers, readers and erasers in crops as well as establishing the molecular link between m^6^A modifications and stress adaptation. Integrating these molecular insights on the regulatory roles of m^6^A modifications in stress response with novel genome‐ and RNA‐editing technologies will pave the way for a novel branch of plant stress biology, which will facilitate the breeding of stress‐tolerant crops via precisely engineered RNA modifications.

## Conflict of interest

The authors declare no competing interests.

## Authors' contributions

H.K. and T.X. conceived the project; H.K., J.H., J.C. and T.X. wrote the paper. All authors read and approved the manuscript.
